# Book Reviews

**Published:** 1822-03

**Authors:** 


### Monthly Medical and Scientific Bibliography.

Vexat censura . . . . . . Corvos.

### 1. GREAT BRITAIN.

Art I.—*A Letter to* Charles Henry Parry, m.d. f.r.s. &c. &c. *on the Influence of Artificial Eruptions, in certain Diseases incidental to the Human Body, with an Inquiry respecting the probable advantages to be derived from further experiments.* By Edward Jenner, Esq m.d. l.l.d. f.r.s. m.n.i.f. &c. &c. &c. and Physician Extraordinary to the King. 4to. pp. 67. Baldwin, and Co. London. 1822.

THE illustrious author of the publication before us, has been long impressed with the idea, "that every pimple with a vesiculated head has an errand to perform for the benefit of the constitution." The influence of pustular eruptions, whether naturally or artificially excited, in many diseases incidental to the human body, can only be despised by superficial observers. To those practitioners who are intimately acquainted with the important functions performed by the dermoid system, the consense existing between it, and the more important organs in the economy, is a fact so striking, that we often find them grounding upon it, their judgment as well as treatment of particular diseases. What wonder, then, that he, who has outstripped all his contemporaries in the consideration of a subject of such high importance, should now come forward and propound, for the curious reflection of the medical public, a question, once keenly agitated, lately neglected, and now again likely to be discussed.

It is well known that the application of tartar emetic to the skin produces a pustular eruption not unlike that of mature small pox—nor can it be wholly forgotten that, at one period, this sort of external application had been recommended, on the false principle of counter-irritation, for the removal of obstinate internal complaints. So far back as the year 1794, Dr. Jenner had directed his attention to this subject; but his experience, at that time, was much too confined to enable him to bring forward facts of sufficient interest for public information; that experience has since been extended, and Dr. Jenner, deeming the question to be one of importance, has brought together several cases which have either passed under his own eye, or those of others on whose testimony he can rely, in order to give it additional interest, and with a view to direct to it an appropriate share of public attention.

The subject seems to have formed, occasionally, the topic of conversation between Dr. Jenner and Dr. Charles Parry—and to the latter, he, therefore, addresses his dissertation in the form of a letter.

The first portion of the volume contains the details of eighteen cases, the results of which, as corroboratory of the efficacy of artificial eruptions in curing various complaints, are most satisfactory.

Case 1, Mania. Cerate with tartarized antimony rubbed on the inside of arms night and morning, which produced papulæ of some magnitude discharging a serous fluid about the third day. "The transition from derangement to health was almost inconceivable."—Case 2, Idiotcy, with a carbuncular tumour between the scapulæ. Tartar emetic ointment applied to the nape of the neck, and the patient regained a sane state of mind soon after the eruptions were produced.—Case 3, Phthisis with anasarca and an enlargement about the left side of the thorax. The tartar emetic ointment rubbed on this part until pustules were produced. Complete cure in six weeks.—Case 4, Spasmodic asthma. Tartar emetic ointment on the nape of the neck. Greatly relieved.—Case 5, Disordered state of the brain; inflammation of the right eye. Tartar emetic ointment rubbed on the left arm. Cured in THREE DAYS.—Case 6, Chronic hepatitis with enlargement and induration. Tartar emetic ointment followed by pustules. Recovered with *astonishing rapidity.*—Case 7, Diaphragmitis—difficult deglutition. Spontaneous eruptions, with discharge, on the legs. Progressive recovery.—Case 8, Hemiplegia, chorea, convulsive fits. Tartar emetic ointment in the line of the cervical vertebræ. Cured.—Case 9, Mania. Tartar emetic ointment employed. Cured.—Case 10, Mania puerperalis. Tartar emetic ointment inside the arm followed by eruption after the lapse of a fortnight. Recovered.—Case 11, Hypochondriasis—mania. Tartar emetic used with much advantage; but the use of it relinquished by the parents before the recovery was complete.—Case 12, Hypochondriasis. Tartar emetic ointment on the pit of the stomach. Recovered.—Case 13, Pyrosis, vomiting of blood. Tartar emetic ointment inside of the arms. Recovered.—Case 14, Hysteria gliding into insanity. Tartar emetic ointment rubbed on the arm. Recovered.—Case 15, Hypochondriasis bordering on insanity. Tartar emetic ointment. Greatly relieved.—Case 16, Hemoptoe. Tartar emetic ointment on the chest. Relieved.—Case 17, Pulmonary affection. Tartar emetic ointment on the chest. Great relief.—Case 18, Intense dyspepsia with pain. Tartar emetic ointment followed by complete recovery.

Such is the summary of the evidence brought forward by Dr. Jenner in support of his favourite plan; and it must be acknowledged to be of a very decided character.

Dr. Jenner next proceeds to comment upon a practice of which he ably traces the history; and after hazarding a few physiological hint upon it, concludes with recapitulating the principal points of inquiry.

This letter contains the abstract of communications from Messrs. Fry, and Fosbrooke, and from the author's own relative, the reverend A. Jenner, speaking favourably of the effects of tartar emetic on the skin in various diseases.

We have long been in the habit of using this remedial agent in obstinate diseases of children on a very extended scale, as the registers of the Royal Metropolitan Infirmary for Children will prove. We know of no other application that will so effectually subdue mesenteric disease, as the one under consideration. Our plan, however, is not to order unctions, which, being tedious operations, and, necessarily, to be repeated frequently, are seldom attended to by the parents: but we direct a soft cerate containing soap or ammoniacum, the surface of which is entirely covered with finely pulverized tartarized antimony, to be applied to the abdomen or scrobiculum cordis, and have thus seldom failed to produce large crops of pustules filled with a thick yellowish, laudable pus. We have adopted the same practice, and with success, for the relief of diseased action in the cranial brain of children; and in our private practice we have recommended the application of external tartarized antimony, with great benefit, in a case of gastrodynia that had resisted every other medicine,—in another of hip disease,—and in a third of hepatic phthisis in which the life of the patient had been despaired of by two other physicians; but where the appearance of the pustules ensured a recovery. The last case we attended with Mr. Willis of Kennington.

---

ART. II.—*A Familiar Treatise on Disorders of the Stomach and Bowels, Bilious and Nervous Affections; with an Attempt to correct many prevalent Errors in Diet, Exercise, &c. being an Exposition of the most approved Means for the Improvement and Preservation of Health.* By GEORGE SHIPMAN, Member of the Royal College of Surgeons in London. 8vo. pp. 172. Sherwin and Co. London.

WE have had this volume by us so long, that we have been tempted to pass it over unnoticed altogether: and, had we followed this plan, the author of it would probably have had reason to thank us. As there are people, however, who prefer being abused, to not being talked of at all: we have, at last, determined on affording Mr. Shipman some degree of celebrity.

Mr. Shipman begins by stating, that as a *minute* anatomical and physiological disquisition tends only to perplex and confuse the mind, he shall only limit his account of the structure of the stomach, and of digestion, to the plainest description and definition. He then proceeds to tell us that the stomach is "a large leathern bottle" in shape, with two openings, containing from three to four pints, composed of membranes, or coats of a peculiar structure, numerously supplied with nerves "from each nervous system," (he does not say from *how many* of the latter)—that the blood vessels, dispersed over it, are numerous; and quite wonderful the ramification of the arteries on its inner surface, where *these arteries secrete the gastric juice*, which is a fluid resembling saliva possessing very *resolvent* properties!" This is anatomy. The physiology of digestion, or the changes to which alimentary matter is subject, is next described with equal precision, and in the same familiar language. We have next some observations on the relative properties of animal and vegetable substances; in mentioning which, we cannot help complimenting the author on the very sensible manner in which he has answered every proposition of Sir Richard Phillips, in support of the universal system of vegetable diet. Some very appropriate remarks on the most prevailing habits of life follow in succession; but we neither met with any thing that is new, nor do we perceive that Mr. Shipman has succeeded in applying the truth of some well-known apophthegms to the removal or better treatment of stomach complaint. Every thing that has been said on diet, Mr. Shipman has repeated in his section, or chapter, entitled "an enquiry into the nutritive property of various kinds of food;" and we confess we have looked in vain, through the concluding part of his volume, for the smallest particle of practical information.

But we have a much more serious ground for reproof against Mr. Shipman; and that is his having, in every instance in which he states to have effected a cure, concealed the nature of the medicines he has given, or stated them only in such general desultory, yet *alluring*, manner, that, while no information can arise to the practitioner who may chance to look into Mr. Shipman's book, patients, for whom it has been familiarly written, may, on perusing it, be induced to send for the very clever man who has performed so many cures by "appropriate medicines" only known to himself. Of course, we are the last persons to attribute such an omission to design; but Mr. Shipman himself, now that we have called his attention to the fact, cannot but admit the justice of our animadversions.

---

Art. III.—*Cases illustrative of the Treatment of Diseases of the Ear, both Local and Constitutional, with Practical Remarks relative to the Deaf and Dumb.* By John Harrison Curtis, Esq. Aurist to His Majesty, and to their Royal Highnesses the Duke and Duchess of Gloucester. 8vo. pp. 94. T. and G. Underwood. 1822.

The profession, we conceive, are indebted to Mr. Curtis for the persevering spirit with which he cultivates the study of those diseases affecting the organ of hearing, which, from their obscurity and intricacy, can only be properly treated by a person entirely devoted to the subject. Having succeeded in establishing a public Dispensary for Diseases of the Ear, which, during a period of nearly six years, has afforded effectual relief to great numbers of the lower classes, Mr. Curtis now brings forward the result of his public and private practice to prove the benefit that deaf persons have derived from his attendance and mode of treatment.

The first subject of a practical nature discussed in this little volume is the collection of matter in the meatus, or passage, termed puriform discharge, constituting one of the most frequent diseases of the ear, and one to which every period of life is liable. Mr. Curtis considers that surgeons were wrong in reporting this complaint as hopeless, and asserts to have found it to yield to proper treatment.

Next to this disease, comes to be remarked that species of deafness which arises from an obstruction of the Eustachian tube; nor is it at all easy, always, to distinguish, whether the imperfection be owing to the cause just mentioned, or be connected with the internal ear.

Mr. Curtis next descants on the important subject of the connexion found to exist between the sense of hearing and the exercise of speech, particularly in children; and immediately afterwards proceeds to the details of his cases, some of which seem particularly interesting.

---

### 2. FRANCE.

**Art. IV.**—*Recherches et Observations, &c. or, Researches and Observations on the effects produced by Preparations of Gold in the treatment of various Diseases.* By J. C. Niel. Published by J. A. Chrestien, of Montpellier.

Gold is one of the metals which, at one time, supplied the mysterious physician with various preparations for the treatment of diseases. Dr. Chrestien has, of late years, taken up the subject in sober earnestness, and wishes us to believe, that some of the oxides and salts of gold are superior in efficacy to many other metallic preparations used in medicine. Some credit is, undoubtedly, due to him for the ingenious methods he has proposed of appropriating gold to the various wants of therapeutics; but how far the practitioners of other countries may be disposed to place confidence in the boasted success which is said to have resulted from the use of that metal, is a question which time alone can determine.

In a work, intitled Jatraleptic Medicine, M. Chrestien had, long ago, published some experiments made with salts of gold, used in frictions; and much of the benefit said to have resulted in his cases, was admitted to have been correctly stated by the author, when his work came under the revision of commissaries appointed by the Institute of France to examine it.

In Hufeland's journal it has been stated, that several German physicians had found the aurific preparations, as recommended by Chrestien, to be most beneficial; and Delafield in America, Gozzi in Italy, and Sorina in Spain, have borne equal testimony to the efficacy of that remedy.

At Montpellier the aurific preparations are held in great estimation; and, if we are to believe the author of the present work (and there are no reasons why we should not), the facts, in support of the utility of M. Chrestien's remedy, are both numerous and strong. The volume before us offers a great variety of these; and, what is greatly in favor of theauthor, contains few or no theoretical reasonings.

M. Niel asserts, that the aurific preparations possess a degree of activity which is not to be found in other medicines, and which appear to exert a specific action on certain diseases, such as tinea, elephantiasis, scrofula, and syphilis. In scrofula, in particular, he contends that they are infinitely superior to mercury; and that they are equally as efficacious as the latter in syphilis.

The aurific preparations excite salivation, which, however, is not attended by any of the unpleasant effects peculiar to the salivation excited by mercury. It is seldom accompanied with inflammation of the buccal membrane, is not fetid, and does not affect the teeth. The only preparations used by M. Chrestien, are the triple muriate of gold and sodium; the oxide of gold precipitated by potash; and gold filings.

A treatment, having for its basis the use of gold, admits of a generous diet and the drinking of wine. It, indeed, renders both highly necessary, since one of the common effects of the aurific preparations is materially to increase the appetite.

In analysing some of the numbers of the *Journal de Pharmacie,* we have, on a former occasion, detailed the processes recommended by M. Chrestien for obtaining the best preparations of gold, mentioned in the present article.

---

ART. V.—*Observations Cliniques, &c.; Clinical Observations, with some general Reflections on Cancerous Affections.* By FELIX MARECHAL, Montpellier, 4to. 1821.

WE have promised to our readers a long article on carcinomatous diseases, and we intend to be as good as our word at a very early opportunity. Till then it will not be amiss to notice the present performance on the same subject:—as we attach so much importance to the consideration of these diseases, we think that too much information cannot be obtained respecting their nature, progress, and treatment.

M. Marechal, like most writers, declares, that he has in vain looked into all the principal works written on cancer, for a clear exposition of the disease. After being perplexed by a chaos of hypotheses on its hereditary and contagious nature, as well as on its presumed causes, he was obliged to close the books in despair, and have recourse to the only genuine fountain of information,—Nature.

The first part of the present work is entirely consecrated to a faithful detail of various cases of the disease under consideration, both real and presumed. The second contains several arguments and analogical inferences, respecting the real nature of the complaint, as deduced from the preceding facts.

In perusing the first case, however, we were struck with the total absence of any symptom indicative of cancer in the parts of generation. The author himself, indeed, after mentioning the recovery of the patient in this case, states that the disease was, probably, a simple inflammation of the left ovarium. If so, why place the case at all in a treatise on cancerous affections?

The four next cases relate to fistulous tumors of the breasts in females, all of which terminated favorably, although in one of them the operation had been recommended by a surgeon of eminence as the only means of saving the patient's life. Low diet, the application of emollient cataplasms, and of leeches, seem to have been the only means resorted to for their treatment.

The sixth and seventh cases are instances of cancerous affection of the testicle, and were cured. We are presented next with two interesting cases of cancer of the lip, in both of which the treatment was successful, and we were struck with the history of a case of *fungus hæmathodes* situated in the internal angle of the right eye, and with that of a cancer or ulcer on the back of the right hand, produced by the rubbing-in a strong caustic remedy on an old wart. In the latter case the patient, who was sixty-six years of age, died, and the author has detailed the appearances on examination of the parts after death. We must refer our readers to the original for an account of the author's views on the above, and many other cases detailed in the second part of the present Essays.

---

### 3. DENMARK.

ART. VI.—*On the Strong Evaporating Property of Camphor, employed as a Remedy in several Diseases.* By Dr. BÖDTCHER. From the Danish Journal, entitled, "Bibliothek for Lœger."

IT is a well-known fact, that camphor possesses a high power of evaporation; and that, when exposed to the open air, it soon diminishes in volume, and ultimately disappears altogether. A curious circumstance, connected with this fact, first directed Dr. Bödtcher's attention to the possibility of its being applied to the purposes of medicine. Happening to enter some years ago the pharmaceutical dispensary of Fridrik's-hospital in Copenhagen, while labouring under a very severe head-ach, some bottles containing camphorated mixture were brought in from the laboratory, and set on the ground not far from the doctor, who, after standing by them for a few minutes, found that his head-ach had entirely left him. This induced him to try the effect of the vapour of camphor, in other complaints, and particularly in those where the camphor itself, or any of its preparations, could not be well applied, as, for instance, in complaints affecting the cavities of the nose, the throat, and chest. The success, said to have followed this new mode of employing camphor as a medicine, is most encouraging. In the worst cases of complete stoppage at the nose, (snuffles,) a piece of camphor need only be kept for a few minutes before that organ, and the patient will, in a short time, be relieved. In inflammation of the eyes it has also proved useful, when held at intervals before them. In cynanche, kept either before the open mouth or the nose, it has produced the best effects. As gargles with camphor, however useful in this complaint, are not always admissible, the knowledge of the fact mentioned by D. Bödtcher may be of great service to practitioners. In the latter disease, the vapour of warm water might be directed to be inhaled with some camphor previously mixed in it. The vapour of camphor has been found very beneficial in common and spasmodic coughs, and even in croup it would be of use, if resorted to in time. The same observation may be made with regard to all kinds of inflammations of the chest.

When gout and rheumatism attack the internal organs, this important remedial agent ought not to be neglected. Experience teaches us that, when camphor is applied to the external parts of the body affected by either of those diseases, the latter will often quit, suddenly, their original seats, and settle, not without great danger, on the internal organs. When this has been the case, the inhaling of the vapour of camphor, Dr. Bödtcher thinks, will be found of great service. In all such cases it would, perhaps, be better to keep a small piece of camphor in the mouth, in order to secure the benefit of its evaporation.

---

### 4. AUSTRIA.

ART. VII.—*Der Geist des Menschen in seinen Verhälmissen zum Physischen Leben, &c. Or, a Review of the Mind of Man in its relation with Physical Life, &c.* By P. C. HARTMAN, Professor of Medicine in the University of Vienna. 1 vol. 8vo. pp. 395. 1820.

THIS is a most singular performance, exhibiting, strongly, the *extravagance* of which a German mind, overflowing with acquired and artificial information, is capable.

The principal or cardinal points of the Professor's scheme, (for we cannot call his system by any other name,) are taken from Professor Wagner's "Ideal Philosophy," published in 1806; and with the addition of the word universe to them, are thus diagramatized:

| | 1. GOD. | |
|---|---|---|
| 2. INTELLIGENCE. (Mind.) | | 3. SUBSTANCE. (Nature.) |
| | UNIVERSE. 4. | |

The author endeavours to shew the catenation which subsists between any two of these entities, and all other functions of the mind and body; striving at the same time to prove, by the most absurd conjectures, that the theory of numbers, and the juxta-position of certain names of faculties and principles, are sufficient to represent, as well as to explain, the complicated *machinery* of the universe.

---

### 5. PRUSSIA.

ART. VIII.—*Addresse à tous les Medecins, &c. Or, an Address to the Members of the Medical Profession of every Country, on the necessity of Preserving the Officinal Names of Medicines.* By Dr. HUFELAND, Counsellor of State, and first Physician to the King of Prussia. Berlin, 1821. 12mo. pp. 28.

THE design of this little pamphlet is to shew the great inconvenience that has arisen from the circumstance of each nation having adopted, in their pharmacopœia, different denominations for the same article, or preparation in the materia medica. Prussia, France, Russia, England, Austria, &c. possess each their pharmaceutical nomenclature; and a druggist, residing in a large capital, where foreigners resort in numbers, is obliged, if he be anxious to understand every prescription that may be sent to him, to collect all the pharmacopœia of those nations, in order that he may consult them when occasion may require.

There was a time when we possessed one intelligible fixed language, common to all nations, for the purpose of directing the compounding of medicines. A prescription, then, written in Berlin, could have been readily made up in any other capital of Europe, or in whatever remote part of the world a chemist's shop was to be found. At present the case is reversed, a patient who is to travel through various dominions must need be provided with as many differently-written prescriptions as there are States through which he is to pass. A prescription written agreeably to the language of the Pharmacopœia of Berlin, observes Dr. Hufeland, has no chance of being understood in Paris, or even in London; and we recollect a prescription of Dr. Baillie giving rise to a sad mistake, in consequence of a Parisian *pharmacien* sending ten grains of solid opium to a patient for whom that very eminent physician had prescribed an equal quantity of extract of poppies.

Dr. Hufeland's apprehensions are by no means exaggerated. We have only to read a few of the examples which he has cited where the same article is differently denominated in different pharmacopœias to perceive that, independently of the great inconvenience which belongs to such a state of things, the danger of mistaking one medicine for another is very considerable. We adduce two or three instances of the confusion of language which prevails when four or five different pharmacopœias are collated together.

Cremor Tartari, in the Austrian Pharmacopœia, is called *Tartris lixivæ acidulus depuratus*—in the Russian, *Supratartras potassæ*—in the Prussian, *Tartarus depuratus*—in the French, *Tartris acidula potassæ.*

Calomel, in the Austrian, *Murias hydrargyri mitis*—in the Russian, *Murias hydrargyri oxydulatus præparatus*—in the Prussian, *Hydrargyrum muriaticum mite*—in the Edinburgh and London Pharmacopœia, *Submurias hydrargyri*—in the French, *Murias hydrargyri dulcis*, or *Protochloruretum hydrargyri.*

Tartarus Emeticus, in the Austrian, *Tartras lixivæ stibiatus*—in the Russian, *Tartras stibii et potassæ*—in the Prussian, *Tartarus stibiatus*—in the English, *Antimonium tartarizatum*—in the Edinburgh, *Tartris antimonii.*

---

### 7. ITALY.

**Art. IX.—Opuscoli Scientifici.** Fascicolo XXIII.—*Cenni intorno al versamento della bile nell' intestino duodeno; &c.*—*Observations on the passage of the Bile into the Duodenum.* By Professor Mondini.

The author thinks that the question as to the precise time when the bile passes into the duodenum is by no means settled; and proceeds to examine the various opinions of Haller, Sabatier, Boyer, Bichat, and Fattori, on this subject. He next enters into a minute and very accurate examination of the structure of the duodenum; and lays a great stress on the fact that this intestine is the only one which is not wholly surrounded by the peritoneum; and notices, in particular, the complicated course which the ductus choledocus takes between the coats of that intestine. Having premised these anatomical facts, the professor undertakes to examine which of the two prevailing doctrines seems more to accord with the structural arrangement of the parts—whether the one which supposes the duodenum in a state of distension when the bile is passing into it; or that which presumes on a state of flaccidity in that intestine during the same operation. The conclusion he draws is in favour of the former doctrine—and the details of some experiments which the professor has made with a view to throw some light on this question, seem to corroborate that conclusion.

His explanation of the physiological fact he has undertaken to discuss is clearly stated in the following passage.

"Dal fin quì detto mi pare potersi inferire, che la bile può, egli è vero, di continuo scorrere dal coledoco nel duodeno in qualunque stato questo si trovi; ma che nella distensione di esso, facendosi il foro del condotto coledoco più manifesto, retta divenendo quella porzione di detto condotto che passa trà le membrane, e accrescendosi per legge vitale l'eccitamento di dette parti la bile perciò si versa in maggior copia nel duodeno; ed al contrario allorchè il duodeno è flaccido, il foro del coledoco è meno manifesto, il dotto stesso forma delle pieghe, minore è l'energia delle parti, ed in conseguenza allora la bile si versa in minor quantità nel duodeno, e rigurgita porzione di essa nella cisti-fellea."

---

Art. X.—*Cenni sull Estirpazione della bocca e del Collo dell' utero, &c. —Observations on the extirpation of the neck and os uteri, in cases of Scirrhus, Cancer, or other morbid Excrescences, with a description of the Pica, a new Instrument for performing the said extirpation with facility and security.* By Giuseppe Cannella, Doctor in Surgery. 8vo. pp. 43. Milan, 1821. Plates.

This essay begins with a rapid sketch of the opinions of Osiander, Manzoni, Dupuytren, and others, on the propriety of performing the operation described in the above title, and agrees with the majority of them, that it can be performed with perfect safety.

Recamier, one of the physicians of the Hotel Dieu in Paris, invented a new speculum uteri for the purpose, principally, of enabling him to apply caustic substances to the cancerous portion of the womb, without at the same time injuring the surrounding parts. Our author having had occasion to use the speculum in question, and apply the nitrate of silver with the assistance of it, to an incipient cancer of the os uteri, in the case of a lady aged 50 years, observed, during the use of the instrument, as well as throughout the period of employing the caustic, that the patient suffered the most excruciating pains, and that the diseased part was but imperfectly destroyed at the end of some months.

Disappointed at this result, and being convinced that, since the object of the plan proposed by Osiander, Recamier, Dubois, Manzoni, and Dupuytren in such cases was to remove the diseased portion of the womb, the sooner such a removal was effected, the more rapid and certain would be the recovery of the patient—Dr. Cannella sat about devising an instrument by means of which the operation might be performed *tuto et cito*, though not *jucunde*. This instrument he has succeeded in bringing to a degree of perfection that promises fairly to make it an engine of great utility in the hands of a judicious and even moderately dexterous practitioner. Hitherto he has had no opportunity of trying it on any patient afflicted with the disease for which it is intended as a relief; and his opinion of its merits is founded upon several experiments made on the dead body: but, from the description of it, and the inspection of the drawings, we are inclined to believe that it will be found a great acquisition to to the operative obstetrician.

---

### ACADEMICAL PROCEEDINGS,

