# Errata in Last Number

**Published:** 1822-04

**Authors:** 


					?? 253, ? 24 from top, instead ot Pita, read Metrotome.

				

